# Multicentric Giant Cell Tumor of Bone: Synchronous and Metachronous Presentation

**DOI:** 10.1155/2013/756723

**Published:** 2013-09-11

**Authors:** Reiner Wirbel, Frank Blümler, Dirk Lommel, Guido Syré, Veit Krenn

**Affiliations:** ^1^Department of Trauma, Hand and Reconstructive Surgery, Verbundkrankenhaus Bernkastel-Wittlich, Koblenzer Street 91, 54516 Wittlich, Germany; ^2^Institute of Diagnostic Radiology, Verbundkrankenhaus Bernkastel-Wittlich, Koblenzer Street 91, 54516 Wittlich, Germany; ^3^Strahlentherapie Trier, August Antz Street 21, 54293 Trier, Germany; ^4^Medical Center of Histology and Molecular Diagnostics, Max-Planck-Street 18, 54296 Trier, Germany

## Abstract

A 27-year-old man treated 2.5 years ago for synchronous multicentric giant cell tumor of bone located at the right proximal humerus and the right 5th finger presented now with complaints of pain in his right hip and wrist of two-month duration. Radiology and magnetic resonance revealed multicentric giant cell tumor lesions of the right proximal femur, the left ileum, the right distal radius, and the left distal tibia. The patient has an eighteen-year history of a healed osteosarcoma of the right tibia that was treated with chemotherapy, resection, and allograft reconstruction. A literature review establishes this as the first reported case of a patient with synchronous and metachronous multicentric giant cell tumor who also has a history of osteosarcoma.

## 1. Introduction

Giant cell tumors (GCT) of bone account for 4% to 5% of all primary bone tumors [1–3]. Multicentricity of GCT of bone is an extreme rarity accounting for less than 1% of these tumors. The biological behaviour and the clinical presentation (e.g., localization, age, and gender distribution) seem to be different from solitary lesions [1–3]. We like to present an additional case of a patient with multicentric giant cell tumor of bone. The typical features of multicentricity should be pointed out.

## 2. Case Presentation

A 27-year-old man presented in March 2012 with pain in his right hip and right wrist of two-month duration. Physical examination revealed no restriction in range of motion to either joint, but localized tenderness was present in the right medial thigh and the dorsoradial region of the right wrist. 

Eighteen years prior, the patient was treated for osteosarcoma of the proximal tibia with chemotherapy and with resection and reconstruction using allograft. The followup included regular clinical and radiographic examination. Treatment was considered successful after ten years without recurrence.

**Figure 1:**
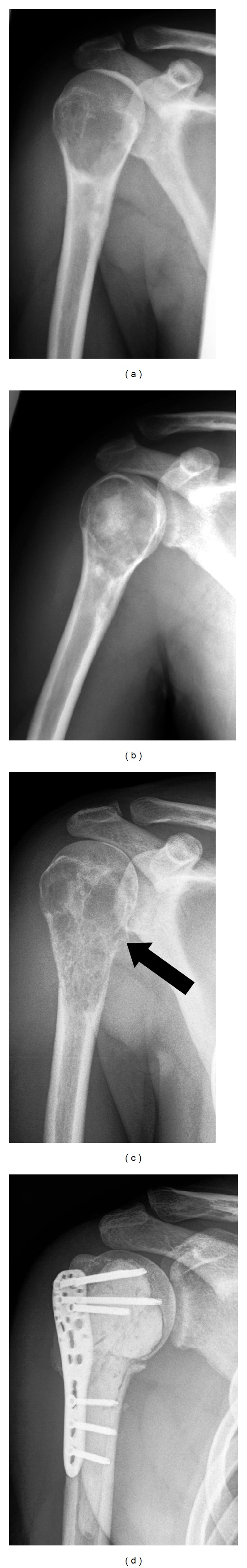
Conventional radiography, AP view of the proximal right humerus: (a) lytic lesion of the proximal humerus, (b) after curettage and filling the defect with cancellous bone graft, (c) presenting with pathological fracture (arrow) four months later, and (d) the lesion was curetted and the cavity was filled with bone cement.

Three years prior, the patient has a lytic lesion of the right proximal humerus (Figure 1(a)) and open biopsy, it was determined to be a giant cell tumor. The lesion was curetted and the cavity was filled with bone graft (Figure 1(b)). Two months later, a biopsy of a swelling of the distal phalanx of the right 5th finger was diagnosed as a giant cell tumor by our Histology Department. Amputation of the distal phalanx was performed. The patient fell two months later after the phalanx amputation resulting in a pathologic fracture of the right proximal humerus (Figure 1(c)). Treatment included curettage of the right proximal humerus and filling the cavity with bone cement (Figure 1(d)). Histological examination confirmed the diagnosis of synchronous multicentric giant cell tumor of bone. Whole-body technetium bone scan showed no further lesions. Follow-up, semiannual, clinical, and radiographic examinations revealed no signs of local recurrence.

**Figure 2:**
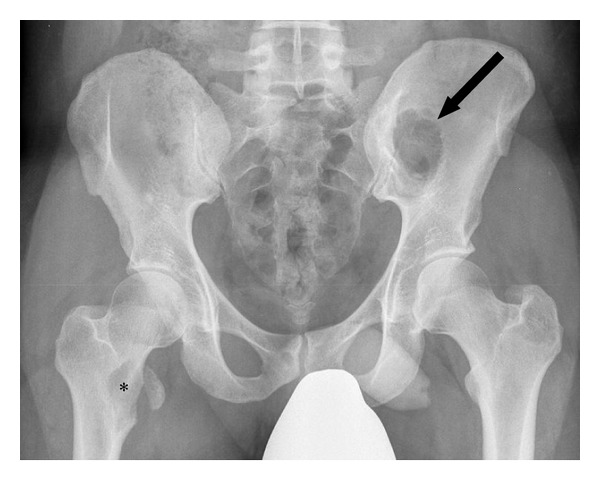
Conventional radiography, AP view of the pelvis: lytic lesions of the right proximal femur (asterisk) and of the left ileum (arrow) became obvious.

**Figure 3:**
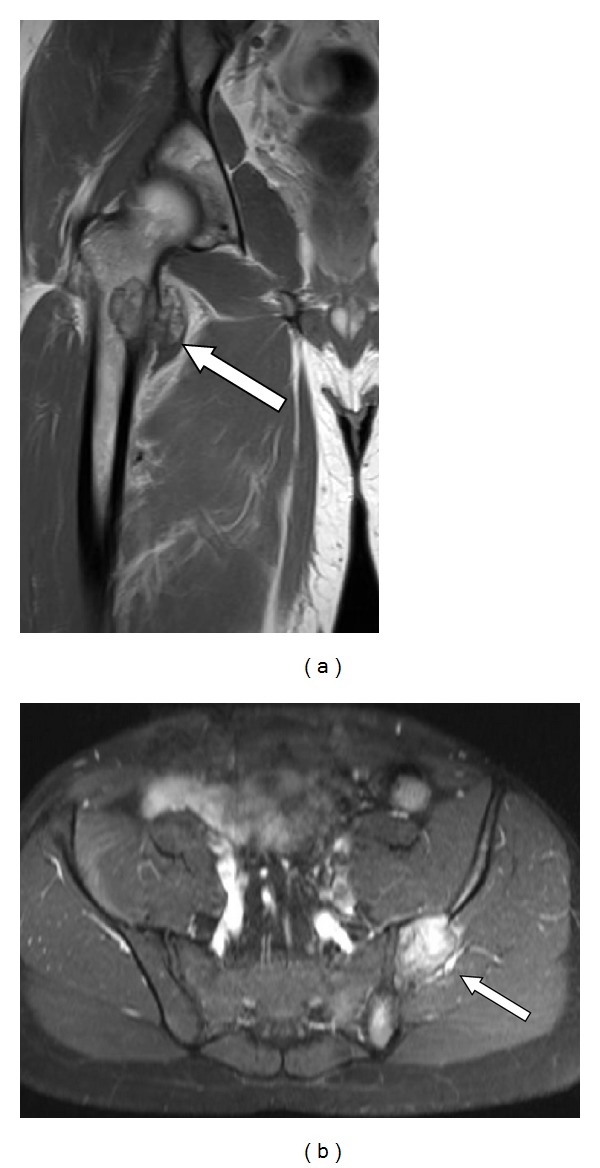
Magnetic resonance imaging: (a) coronal image of the right proximal femur shows the soft tissue involvement (arrow) of the lesser trochanteric region, and (b) axial image of the pelvis demonstrates the soft tissue involvement of the right gluteal region (arrow) nearby the iliac wing and the sacroiliac joint.

Radiographic examination of the patient revealed lesions in both painful areas. Plain radiograph of the pelvis (Figure 2) revealed a typical 3 cm lytic lesion involving the right proximal medial femur and a 4 cm lytic lesion involving the left ileum. MRI of the pelvis (Figure 3) showed the extension of the lesions with soft tissue involvement of the right thigh and of the left gluteal region. 

**Figure 4:**
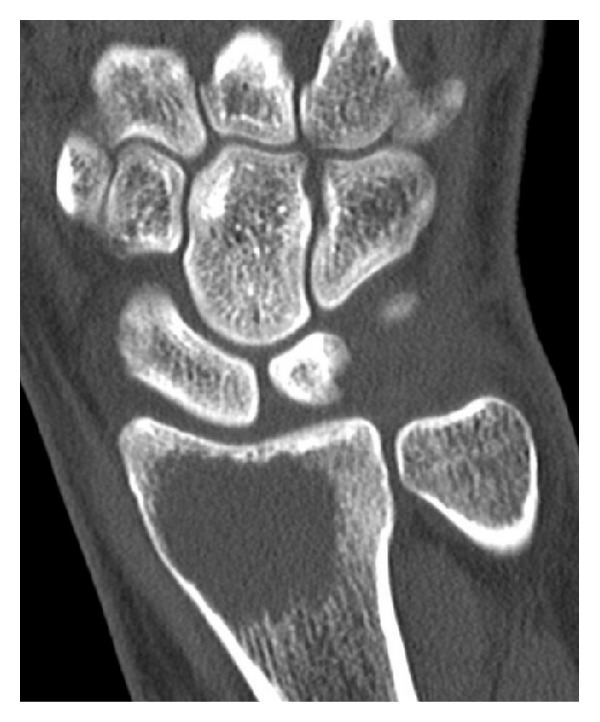
Computed tomography of the right wrist (coronal section): the extension of the lytic lesion in the subchondral region of the distal radius is well recognized.

Plain radiograph and CT of the right wrist (Figure 4) revealed a 4 cm lytic lesion involving the epiphyseal subchondral region of the distal radius but without cortical breach. 

**Figure 5:**
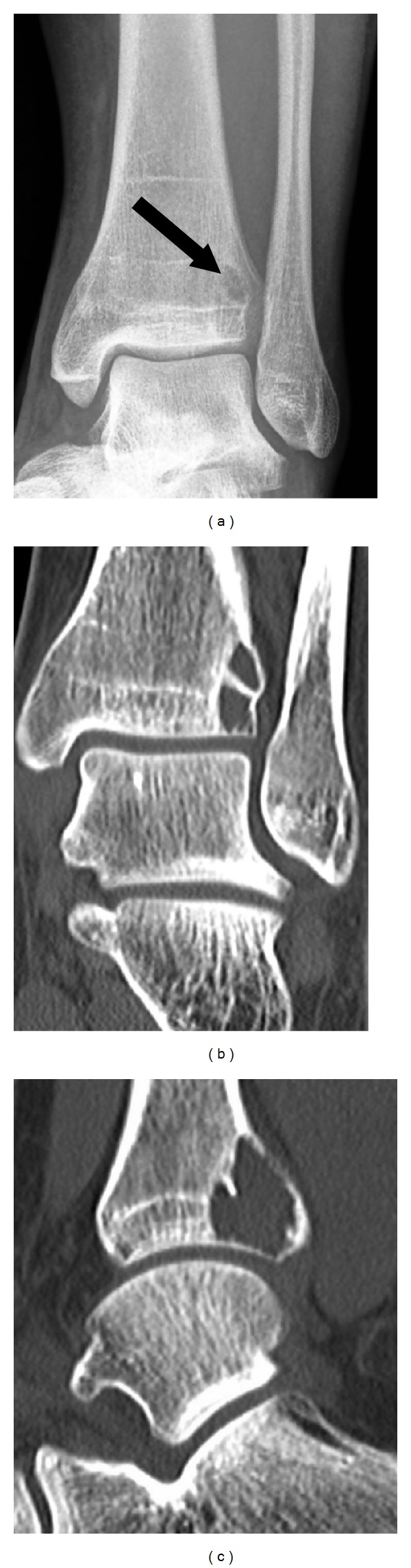
Conventional radiography and computed tomography of the left distal tibia: (a) plain film (AP view) presents the lytic lesion (arrow) of the left lateral distal tibia, and (b) coronal and (c) sagittal CT sections can recognize the exact extension of the subchondral distal region of the dorsolateral left tibia.

Further radiographic examinations were performed to rule out the presence of other lesions or metastasis. The whole-body technetium bone scan revealed an additional increased uptake in the left distal tibia. The plain radiograph (Figure 5(a)) and the CT (Figures 5(b) and 5(c)) of the left ankle joint showed a further 2 cm lytic lesion of the subchondral region of the distal dorsolateral tibia. CT of the thorax and abdomen confirmed the absence of metastasis. 

To rule out hyperparathyroidism acid and alkaline phosphatase and serum calcium were within normal range. Serum parathyroid hormone level was also normal. 

**Figure 6:**
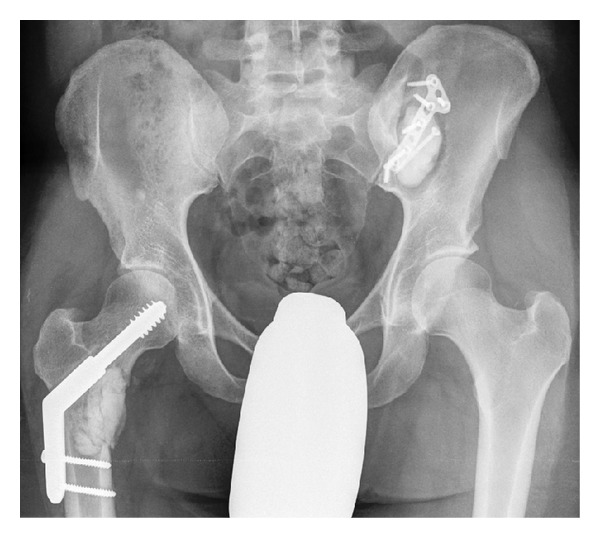
Conventional radiography of the pelvis (AP view): after curettage of the lesions at the right proximal femur and of the left iliac wing, the defects were filled with bone cement; prophylactic stabilizations using a dynamic hip screw at the right side and a plate at the left posterior iliac crest were performed.

All lesions were removed using powered burrs and were chemically cauterized with phenol solution. The resulting bone cavities were filled with bone cement. Prophylactic bone stabilization was performed in all areas where lesions were removed. A dynamic hip screw was used to stabilize the right hip; a plate was used to fix both the left posterior iliac crest (Figure 6) and the right distal radius; a single screw was adequate to fix the left distal tibia. The postoperative course was uneventful with full weight bearing was possible within two weeks. 

**Figure 7:**
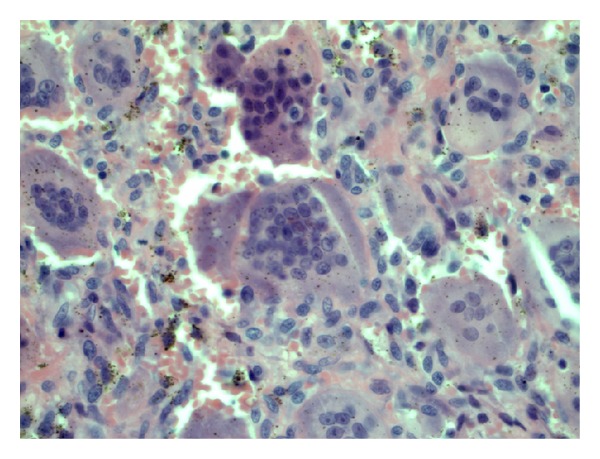
Microscopic pathology: H-E stain at 200x at the lesion of the right femur: the specimen demonstrated large osteoclasts and uniform mononuclear cells with ovoid nuclei, giant cells containing about 50 to 100 nuclei, and ill-defined cytoplasm with very few intercellular collagen.

Histological examination of all lesions revealed the typical findings of a giant cell tumor of a bone (Figure 7). The tissue was composed of large osteoclasts and uniform mononuclear cells with ovoid nuclei. The giant cells contained 50 to 100 nuclei. The nuclei of the stromal cells were similar to the nuclei of the osteoclasts having a so-called open chromatin pattern with indistinct nucleoli. The cytoplasms were ill-defined with very few intercellular collagen, broad sinus, and retraction artefacts. Mitotic figures were very rare. 

Radiation therapy was recommended for the two pelvic lesions, by our multidisciplinary tumor board, because of soft tissue involvement.

Informed consent was taken from the patient prior to operation for the procedures as well as using his clinical data in this case report.

## 3. Discussion

A literature review was undertaken to determine the prevalence of synchronous and metachronous, multicentric giant cell tumor of bone. Giant cell tumor (GCT) of bone is a benign primary neoplasm accounting for 4% to 5% of all primary bone tumors [1–4]. GCT of bone typically presents as a solitary lytic lesion of the metaphyseal-epiphyseal region of a long bone of adults [1] and in nearly 50% of the cases they occur adjacent to the knee joint [3–6]. 

Multicentric GCT of bone is extremely rare and accounts for less than 1% of GCTs [1–3]. Approximately 100 cases have been reported worldwide, mostly single case descriptions or studies of small series [3–5, 7–15].

Multicentric lesions are considered to occur more often than solitary lesions in younger patients [1–3, 7–9, 11–13] with an average age of twenty years at presentation and 59% of patient younger than twenty [3]. It occurs more often in females (twice as often as in males) and skeletal immature patients [1–4, 9, 11, 13].

Patients usually present with two to three lesions, but one patient with ten tumors has been described [14]. When compared with solitary lesions, multicentric GCT is reported more frequently in the short bones of the hands and feet [3, 4, 8, 9, 13, 15].

Multicentric lesions are considered to be more frequently restricted to the metaphyseal or diaphyseal-metaphyseal region of the long bones than solitary lesions, which predominantly involve the metaphysis and the epiphysis [1–4, 6].

Lesions of multicentric GCT of bone are classified as metachronous or synchronous. When multiple tumors are discovered at the initial presentation or when a second lesion is diagnosed within six months, the lesions are classified as synchronous [1–3]. 

Synchronous tumors occur less frequently than metachronous tumors [1–3, 5]. If a second tumor develops more than six months after the first lesion, the lesions are classified as metachronous. 

In most cases of metachronous GCTs additional tumors are developed within two years after the discovery of the primary lesion; however, they may develop twenty or more years later [10]. This is apparently the first reported case of multicentric giant cell tumor of bone with synchronous and metachronous presentation, since no other cases were present in the literature where both synchronous and metachronous lesions occurred in the same patient.

The pathogenesis of the multicentricity in GCT is still unclear but various mechanisms have been suggested including contiguous spread, iatrogenic seeding of tumor cells, benign metastasis, malignant transformation, and de novo multifocal formation [4, 10, 15].

In most cases of multicentric GCTs diagnosis can be guided by clinical, serological, and radiographic findings. They should be distinguished from other multifocal lesions; such as brown tumors of hyperparathyroidism, multifocal giant-cell reparative granuloma, Paget's disease, fibrous dysplasia, fibrosarcoma, metastasis, osteosarcoma, multiple myeloma, and multifocal osteomyelitis [1, 3, 8, 16].

Radiographic examination using plain film usually demonstrates an ecpansile, excentric radiolucent lesion at the epiphyseal, subchondral region with well-defined geographical margins. In the affected area there is a lack of sclerosis and of internal mineralization and the cortices of the bone are expanded or destroyed. MRI is currently the best imaging modality allowing accurate tumor delineation, extraosseous extent, and articular surface involvement. The tumor mass is isointense in T1 imaging and slightly hyperintense in the T2 imaging with contrast media enhancement. Necrotic areas of the tumor appear hypointense in the T1 but hyperintense in the T2-weighted image [1, 3, 6]. CT is a reasonable alternative especially to define the intra-osseous extension of the tumor [3, 6].

Scintigraphic bone scan usually demonstrates a diffuse increased radionuclide uptake or a peripherally increased uptake and photopenia centrally (“donut” sign) [3].

The histogenetic origin of the GCT of bone is unknown. The histological appearance of the tumor is very variable and consists mainly of giant cells containing multiple nuclei as well as single nucleus stroma cells. Multicentric GCT of bone is histologically indistinguishable from solitary lesion; however, fibroblastic and fibrohistiocytic areas are considered to be a major component of these tumors [1, 3].

Although there are clinical and radiographic similarities between solitary and multicentric lesions, the risk of pulmonary metastases is reported at 5–10% in patients with multicentric GCTs but only at 1-2% in patients with solitary GCTs [1, 3].

The primary goal of treatment of this tumor is to eradicate the lesions and to preserve the function of the affected bones and joints. The preferred treatment method is intralesional curetting and filling the cavity with bone cement [1, 3, 5, 6, 8]. Since the detection of recurrent lesions is enhanced by the use of bone cement it is considered more appropriate than the use of primary bone graft. Thermal cauterization of the bone cement, by its exothermic reaction in combination with local chemical cauterization using phenol may help destroy microscopic tumor cells and reduce the chance of recurrence [1, 5].

Recurrence after intralesional curettage is reported to be 25% whereas wide excision is associated with a rate of 5% [1, 3, 8], but wide excisions usually require endoprosthetic replacement or arthrodesis because of the tumors frequent juxta-articular localization. 

Exclusive radiation therapy is generally ineffective in controlling these tumors, but it is reserved for lesions that are not amenable to surgical removal. Radiation therapy is recommended in patients with soft tissue involvement (after treatment with curettage and bone cement) because they may have an increased risk of local recurrence [1–3, 6, 8].

Malignant transformation of GCT of bone can be defined as a sarcoma, usually a fibrosarcoma or osteosarcoma. No exact data were found in the literature about malignant transformation of multicentric lesions but up to 5% of solitary lesions undergo malignant changes [1, 3]. 

Prior to this case report, the development of multicentric GCT after an osteosarcoma has not been described in the literature. 

This occurrence of multicentric lesions developing after an osteosarcoma and the well-documented tendency of multicentric tumors to affect younger patients [3, 5, 9, 11, 13] suggest that there may be a germ-line genetic abnormality that predisposes these patients to the development of multiple tumors, but familial forms of multicentric GCTs of bone have not been reported [3]. 

Bones scan screening at unusual sites (i.e., diaphyseal region, small bones) is recommended for patients with solitary lesions on a semiannual basis for five years since most cases of multicentricity occur within this period [3].

In patients with GCT of bone multicentricity has to be taken in consideration when the patients are of younger age or when the lesion is located at unusual sites (i.e., diaphyseal region, small bones of the hands or feet). MRI is considered to be the best imaging modality allowing accurate tumor delineation, as well as the soft tissue and articular surface involvement.
